# Immunotherapy for multiple myeloma: new chances and hope

**DOI:** 10.20892/j.issn.2095-3941.2023.0258

**Published:** 2023-09-28

**Authors:** Jingyu Xu, Gang An, Lugui Qiu

**Affiliations:** 1 State Key Laboratory of Experimental Hematology, National Clinical Research Center for Blood Diseases, Haihe Laboratory of Cell Ecosystem, Institute of Hematology & Blood Diseases Hospital, Chinese Academy of Medical Science & Peking Union Medical College, Tianjin 300020, China; 2 Tianjin Institutes of Health Science, Tianjin 301600, China

## Introduction

Multiple myeloma (MM), characterized by the proliferation of monoclonal plasma cells in the bone marrow, has the second highest incidence among hematologic malignancies^1^. Because of its incurable nature, treatments for MM are aimed primarily at obtaining minimal residual disease (MRD) negativity and achieving persistent control, both of which are believed to be important strategies to prolong survival and improve prognosis in patients with MM.

Three-drug regimens containing proteasome inhibitors (PIs) and immunomodulatory drugs (IMiDs), followed by autologous stem cell transplantation (ASCT) and maintenance, have been important standard treatments for MM. Real-world data from 1,000 patients with MM with bortezomib/lenalidomide/dexamethasone (VRD) induction have shown extraordinary results, in which 89.9% of patients achieved very good partial response or better, and the median overall survival (OS) for the entire cohort exceeded 10 years^2^. Other next-generation PIs and IMiDs have also shown good results in clinical studies.

**Figure 1:**
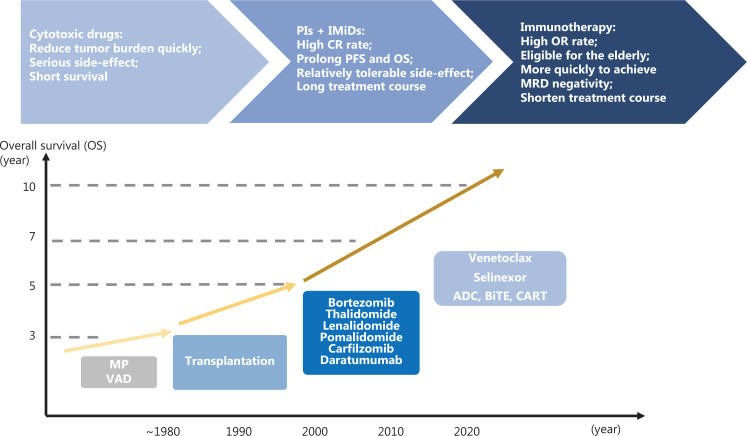
Development of treatments for MM. PIs, proteasome inhibitors; IMiDs, immunomodulatory drugs; CR, complete response; PFS, progression-free survival; OS, overall survival; OR, overall response; MRD, minimal residual disease; MP, melphalan, prednisone; VAD, vincristine, doxorubicin, dexamethasone; ADC, antibody drug conjugates; BiTE, bispecific T-cell engager; CART, chimeric antigen receptor T cell.

However, most patients eventually develop disease progression, characterized by immune escape and resistance to standard therapy, and the tumor environment (TME) plays an important role in this process. In recent years, several novel immunotherapeutic approaches targeting tumor cells and the TME have been developed, thus providing new chances and hope for patients with MM, particularly those previously heavily treated. Here, we review recent advances in immunotherapy and its clinical applications in treatment strategies aimed at achieving persistent MM control (treatment development shown in **Figure 1**).

## Immunotherapy

Several immunotherapeutic approaches for MM have achieved significant results in clinical trials, including monoclonal antibodies (mAbs), chimeric antigen receptor (CAR) T cells therapy, bispecific T cell engagers (BiTEs) and antibody drug conjugates (ADC). There were also some novel noncellular immunotherapies like CELMoDs, immunocytokines, immunotoxins, and NK-cell activators/engagers. Here, we focus our review on cellular immunotherapy.

### mAbs

mAb therapies are directed primarily against antigens expressed on the surfaces of malignant plasma cells (PCs). CD38 is highly expressed on the surfaces of not only PCs but also immunosuppressive cells, such as Bregs, Tregs, and myeloid-derived suppressor cells. Consequently, targeting CD38 can alleviate the immunosuppressive microenvironment^3^. Daratumumab, a fully human immunoglobulin G1-κ (IgG1-κ) antibody, was the first antibody to CD38 administered in MM treatment. Daratumumab-based regimens have been found to increase the depth of response and progression-free survival (PFS) in newly diagnosed multiple myeloma (NDMM) and relapsed/refractory MM (RRMM). Importantly, the GRIFFIN study has demonstrated enhanced benefits of adding daratumumab to VRD as a first-line induction regimen for transplantation eligible patients with MM^4^. Daratumumab has been included in first-line therapy for NDMM. Isatuximab, another IgG1-κ chimeric monoclonal antibody with a mechanism of action similar to that of daratumumab, has also shown good efficacy in the treatment of RRMM^5^.

Elotuzumab is another humanized IgG immunostimulatory monoclonal antibody targeting signaling lymphocytic activation molecule F7 (SLAMF7), a glycoprotein highly expressed on the surfaces of MM cells and natural killer cells^3^. In clinical studies, combination regimens containing elotuzumab for NDMM treatment have not been found to increase treatment efficacy or survival^6^. However, phase II ELOQUENT-3 Trial showed EPd improve both PFS and OS than Pd in patients with RRMM^7^.

### CAR-T cell therapy

B cell maturation antigen (BCMA) is more abundant in malignant PCs than normal PCs^3^. BCMA is activated by a proliferation-inducing ligand (APRIL), and its overexpression augments MM cell growth and survival *via* the induction of MAPK and NF-κB signaling cascades^8^. Consequently, BCMA plays important roles in cell proliferation and drug resistance, and therefore is an ideal target for MM immunotherapy.

Currently, 2 BCMA targeting CAR-T cell therapies, idecabtagene vicleucel (ide-cel) and ciltacabtagene autoleucel (cilta-cel), have been approved by the FDA on the basis of their impressive clinical results in RRMM. The KarMMa study revealed unexpected success of ide-cel in heavily pretreated patients with RRMM^9^. Regarding cilta-cel, the most recent update in 2023 indicated a stringent complete response (CR) in 82.5% of patients, and median PFS and OS had not been reached at a median follow-up of 27.7 months^10^. GPRC5D, an orphan receptor expressed on malignant PCs and hair follicles is another candidate target^11^. In a phase II trial in China, GPRC5D CAR-T therapy has shown encouraging clinical efficacy, with 66% patients obtaining CR or better, and a manageable safety profile in patients with RRMM, including patients with cancer progression after anti-BCMA CAR T-cell therapy^12^.

### BiTEs

Bispecific antibodies are dual-targeting antibodies that bind target antigens on the surfaces of malignant cells and effector cells, and subsequently exert anti-tumor effects by redirecting T cells to tumor cells. Currently, the target antigen on effector cells in nearly all BiTEs is CD3 on T cells; other commonly used targets on tumor PCs include BCMA, GPRC5D, and CD38^3^. BiTEs currently under clinical testing include BCMA/CD3 BiTEs, such as teclistamab and elranatamab, and the GPRC5D/CD3 BiTE talquetamab.

Teclistamab has shown a high rate of deep and durable response in RRMM, with an ORR of 63.0% and a rate of complete remission or better (≥ CR) of 39.4% in the MajesTEC-1 trial^13^. Preliminary results of elranatamab have indicated a manageable safety profile and deep clinical responses in patients with RRMM, even after prior BCMA-targeted therapy; this treatment is under phase I clinical investigation in the Magnetismm-1 study^14^. In the MonumenTAL-1 clinical study, talquetamab has been found to induce a substantial response and to have manageable adverse events in patients with heavily pretreated RRMM^15^.

### ADCs

ADCs offer a new immunotherapy option for patients with MM. These conjugates consist of 3 main components: antibodies that selectively recognize antigens on the surfaces of cancer cells, a small cytotoxic molecule referred to as the payload, and a chemical linker connecting the antibody and the payload. Several BCMA ADCs are under clinical development, among which the most attractive is belantamab mafodotin. The DREAMM-2 clinical trial has indicated this treatment’s anti-myeloma activity and manageable safety profile^16^. However, the failure of the DREAMM-3 clinical trial, comparing single agent belantamab mafodotin to pomalidomide plus low dose dexamethasone, led to the withdrawal of belantamab mafodotin (NCT04162210).

### Sequential immunotherapy

For patients with RRMM, immunotherapy is increasingly becoming the treatment of choice. Even if patients experience disease progression after immunotherapy, changing the target antigens and immunotherapy modalities may be a feasible and effective option. The CARTITUDE-2 (Cohort C) clinical study has achieved successful sequential treatment with cilta-cel in patients with RRMM and prior exposure to anti-BCMA ADC and anti-BCMA BiTE, thus providing new hope and options to optimize outcomes and maximize benefits for heavily treated patients^17^. BiTEs are also effective options for sequential immunotherapy. Updated results on teclistamab in RRMM after exposure to other BCMA-targeted agents have been reported in 2022 ASCO Annual Meeting. However, a real-world multi-center data analysis has revealed that patients with prior BCMA-targeted therapy (BCMA-TT) before ide-cel experience inferior PFS and poorer responses than patients without prior BCMA-TT. Moreover, this effect is particularly pronounced in patients with an interval of <6 months between BCMA-TT and ide-cel treatment^18^.

Consequently, in sequential therapy, clinicians need to be aware of the relevant factors, including a shorter duration of the last anti-target treatment and a longer interval between immunotherapy sessions^17,18^. Overall, sequential therapy is beneficial in prolonging patient survival and outcomes, but more data are needed regarding the interval between immunotherapy sessions and the choice of immunotherapy modality.

## Combination therapy with immunotherapy

Although CAR-T has shown significant efficacy in RRMM treatment, not all patients achieve a response, and the short remission time and high relapse rate after CAR-T therapy limit long-term survival. Consequently, clinical studies are increasingly integrating immunotherapy into overall treatment systems, such as by combining it with conventional treatments as a first-line therapy. The main areas being explored in CAR-T trials are (1) options for patients who are not candidates for transplantation; (2) complementary salvage therapy for patients with poor outcomes after treatment; and (3) combination with conventional therapy and transplantation to improve prognosis. The CARTITUE, KarMMa, and MagnetisMM clinical trials are the best examples, as listed in **Table 1**. CARTITUDE-2 was designed for different target patients and has indicated excellent results in patients with early relapse, with a CR rate exceeding 90%^19^. Moreover, promising results from KarMMa-2 Cohort 2a and Cohort 2c were reported at 2022 ASH Annual Meeting, indicating a deep response and excellent MRD negativity rate.

**Table 1:** Clinical trials integrating immunotherapy into overall treatment systems

Study	NCT number	Treatment	Disease status	Results
CARTITUDE-2	NCT04133636	Cilta-cel	Cohort A: PD after 1–3 prior lines of therapy	mFU 17.1 m, ORR 95%, MRD negativity rate 100%
			Cohort B: early relapse after front-line therapy	mFU 18.0 m, ORR 100%, MRD negativity rate 93%
			Cohort C: RRMM after PI, IMiD, anti-CD38, and anti-BCMA therapy	mFU 11.3 m, ORR 95%, MRD negativity rate 70%
			Cohort D: NDMM after first-line induction therapy and ASCT	/^#^
			Cohort E: HRNDMM	/^#^
			Cohort F: NDMM with very good partial response or better after initiation of therapy	/^#^
CARTITUDE-5	NCT04923893	VRd followed by cilta-cel *vs.* VRd followed by Rd	NDMM; ASCT not planned as initial therapy	Recruiting^*^
CARTITUDE-6	NCT05257083	Dara-VRd followed by cilta-cel *vs.* Dara-VRd followed by ASCT	NDMM	Not yet recruiting^*^
KarMMa-2	NCT03601078	Ide-cel	Cohort A: PD within 18 months of initial treatment with ASCT	mFU 21.5 m, ORR 83.8%, MRD negativity rate 70%
			Cohort B: PD within 18 months of initial treatment without ASCT	mFU 17.8 m, ORR 100%, MRD negativity rate 93%
			Cohort C: MM with inadequate response post ASCT during initial treatment	mFU 27.5 m, ORR 87.1%, MRD negativity rate 62.9%
KarMMa-4	NCT04196491	4 cycles of induction therapy followed by ide-cel	HRNDMM	/^#^
MagnetisMM-6	NCT05623020	Elranatamab+DR *vs.* DRd	NDMM; ineligible for transplantation	Recruiting^*^
MagnetisMM-7	NCT05317416	Elranatamab *vs.* lenalidomide	NDMM with MRD positivity after transplantation	Recruiting^*^

^*^From ClinicalTrials.gov. ^#^No available data. mFU, median follow-up.

Combining immunotherapy with conventional therapies to achieve additive effects greater than those of the constituent therapies remains a challenge. In incorporating immunotherapy into the overall treatment system, the timing and target patients for applying immunotherapy must be considered. MRD negativity is among the most powerful prognostic indicators. However, only a subset of patients achieve MRD negativity after ASCT or first-line therapy, and residual plasma cells are susceptible to resistance to previous regimens; therefore, integrating multiple treatment options to help high-risk patients achieve MRD negativity is key to prolonging their survival. Consequently, choosing immunotherapy as the first-line therapy or when patients do not obtain MRD negativity may be a reasonable option to enhance treatment response. Another target group is older people who are ineligible for transplantation or who cannot tolerate the adverse effects of conventional chemotherapy because of their frailty. Under these circumstances, immunotherapy is safe and effective, and it can also shorten the course of conventional treatment and improve patient quality of life. In addition, other high-risk plasma cell disorders, such as plasma cell leukemia and systemic light chain amyloidosis, also need to be concerned.

Moreover, in the era of immunotherapy, the role of ASCT and the combination of ASCT and immunotherapy must be reevaluated. Several studies have shown that ASCT augments CAR-T efficacy. The application of ASCT before CAR-T can decrease tumor cells in the bone marrow, modulate the TME, and remove immunosuppressive factors that impede CAR-T functioning. In addition, infusing CAR-T cells after ASCT can further remove residual tumor cells, thus prolonging PFS. Regimens of ASCT combined with CAR-T have yielded favorable results in several clinical studies^20^.

Clinical trials are increasingly indicating the importance of immunotherapy and are attempting to discover more possibilities in immunotherapy. Future data may provide powerful evidence of whether patients in early disease stages may benefit from these strategies.

## Conclusions

The era of immunotherapy has arrived, and the efficacy of immunotherapeutic treatments has been verified in clinical practice. Ongoing areas are setting immunotherapy to the first-line treatment landscape to achieve deep response and prolong survival. Furthermore, deciphering mechanisms of resistance to immunotherapeutic strategies, regulating the TME, and reversing immunosuppression of the microenvironment will be essential to achieve clinical benefits.
